# Improved high-throughput screening technique to rapidly isolate *Chlamydomonas* transformants expressing recombinant proteins

**DOI:** 10.1007/s00253-022-11790-9

**Published:** 2022-02-07

**Authors:** Ashley E. Sproles, Anthony Berndt, Francis J. Fields, Stephen P. Mayfield

**Affiliations:** 1 The California Center for Algae Biotechnology, University of California, San Diego, La Jolla, CA USA; 2 Division of Biological Sciences, University of California, San Diego, La Jolla, CA USA

**Keywords:** Microalgae, Fluorescence activated cell sorting (FACS), Biotechnology, Bioproducts

## Abstract

**Abstract:**

The single-celled eukaryotic green alga *Chlamydomonas reinhardtii* has long been a model system for developing genetic tools for algae, and is also considered a potential platform for the production of high-value recombinant proteins. Identifying transformants with high levels of recombinant protein expression has been a challenge in this organism, as random integration of transgenes into the nuclear genome leads to low frequency of cell lines with high gene expression. Here, we describe the design of an optimized vector for the expression of recombinant proteins in *Chlamydomonas*, that when transformed and screened using a dual antibiotic selection, followed by screening using fluorescence activated cell sorting (FACS), permits rapid identification and isolation of microalgal transformants with high expression of a recombinant protein. This process greatly reduces the time required for the screening process, and can produce large populations of recombinant algae transformants with between 60 and 100% of cells producing the recombinant protein of interest, in as little as 3 weeks, that can then be used for whole population sequencing or individual clone analysis. Utilizing this new vector and high-throughput screening (HTS) process resulted in an order of magnitude improvement over existing methods, which normally produced under 1% of algae transformants expressing the protein of interest. This process can be applied to other algal strains and recombinant proteins to enhance screening efficiency, thereby speeding up the discovery and development of algal-derived recombinant protein products.

**Key points:**

• *A protein expression vector using double-antibiotic resistance genes was designed*

• *Double antibiotic selection causes fewer colonies with more positive for phenotype*

• *Coupling the new vector with FACS improves microalgal screening efficiency* > *60%*

**Supplementary Information:**

The online version contains supplementary material available at 10.1007/s00253-022-11790-9.

## Introduction

Microalgae, which include photosynthetic eukaryotes and cyanobacteria, hold great potential as a source of commercial bioproducts, and are currently gaining popularity in biotechnology applications. The cultivation of microalgae is attractive due to its enhanced sustainability over alternative sources, where it alleviates environmental issues surrounding land usage, greenhouse gas emissions, and global food demands (Herrero and Ibáñez 2015). Algae can be grown on non-arable land in open ponds or in bioreactors using non-potable water, and have the potential to directly sequester CO_2_ from power plant flue gases to reduce carbon emissions (Khoo et al. 2013; Cheah et al. 2014). Their biological features allow cultures to quickly reach high densities, producing larger quantities of biomass per area than plants, and can use resources that do not compete with food crops (Dismukes et al. 2008; Herrero and Ibáñez 2015; Moreno-Garcia et al. 2017).

Historically, the interest in cultivating microalgae at large scale was spurred by global environmental concerns over fossil fuel emissions, leading to the development of algae as a feedstock for biofuels (Sheehan et al. 1998). However, it quickly became clear that to allow economic viability of algal biofuels, valuable co-products would be required, to offset production costs (Stephens et al. 2010; Wijffels and Barbosa 2010; López Barreiro et al. 2014). Investigations into potential co-products has identified many high-value naturally derived compounds, including proteins, oils, carbohydrates, pigments, and small molecules, that can be sold in a variety of markets, such as specialty chemicals, nutritional supplements, feeds and foods, cosmetics, and pharmaceuticals (León-Bañares et al. 2004; Borowitzka 2013; Chew et al. 2017).

In addition to naturally derived products, some microalgae species have been successfully genetically engineered to modify metabolic pathways or express high-value recombinant proteins. To date, there have been over 40 different therapeutic proteins expressed in algal chloroplasts, with many being viral and bacterial antigens that can be used as vaccines for humans and animals (Dyo and Purton 2018), while many other pharmaceutical products are under investigation (Specht and Mayfield 2014; Kwon et al. 2019; Sproles et al. 2021). Furthermore, algae have been engineered to produce proteins for use as human and animal health supplements, as well as industrial enzymes (Georgianna et al. 2013; Manuell et al. 2007; Rasala and Mayfield 2015). *C. reinhardtii* is also known to be safe for ingestion, and shown to have beneficial effects on the gastrointestinal health of animals and humans (Murbach et al. 2018; Fields et al. 2020), thus an ideal candidate for producing edible recombinant proteins.

Genetic tools for the green microalga *Chlamydomonas reinhardtii* have been developed over the last few decades, allowing this species to become a model expression system for bioengineering of high-value products (Merchant et al. 2007; Scranton et al. 2015; de Carpentier et al. 2020). All three of the *C. reinhardtii* genomes—nuclear, chloroplast, and mitochondria—have now been sequenced and annotated (Vahrenholz et al. 1993; Maul et al. 2002; Merchant et al. 2007), and all are amenable to genetic transformation through various methods (Jinkerson and Jonikas 2015; Kindle et al. 1991; Remacle et al. 2006). To date, expression of recombinant proteins has mainly been reported from transgenes inserted in the chloroplast genome, due to the relatively facile homologous recombination in that organelle, that allows site-directed gene insertion, as well as strong, well characterized endogenous promoters, and eukaryotic-like protein folding cellular machinery that allows resulting proteins to be expressed at 0.2–5% of total soluble protein (Manuell et al. 2007; Mayfield et al. 2007). In contrast, engineering of the nuclear genome has been more challenging, obtaining much lower recombinant protein accumulation—with reported maxima of only 0.2% of total soluble protein—due to factors such as positional effects from random integration of genes and associated local chromatin effects, RNA silencing, as well as potential epigenetic suppression of transgenes (Neupert et al. 2009; Potvin and Zhang 2010). However, chloroplast engineering comes with the drawback of often using light-regulated promoters, and lacking the mechanisms for post-translational modifications such as glycosylation, which is important for many therapeutic proteins (Eichler-Stahlberg et al. 2009; Lingg et al. 2012; Mayfield et al. 2007). Nuclear-integrated transgene expression is therefore more ideal and experiences more stable transgene expression (Harris 2009). Because of these factors, the improvement of nuclear recombinant protein expression is a major focus of algae biotechnology research.

Currently, the most significant bottleneck in this bioengineering process is screening for clones with high expression of the desired product, since recombinant genes are integrated randomly into the genome (i.e., expression is heavily influenced by positional effect). Screening typically involves using a drug resistance gene in the expression cassette, so that algae can first be selected on agar plates containing the appropriate antibiotic (Neupert et al. 2012), and then subsequently screened for recombinant protein accumulation in the resulting colonies. Because only a small percentage of drug-resistant colonies has the potential for high level of recombinant protein production, this protein screening process can be relatively lengthy and time-inefficient as most methods require bulk DNA extraction, PCR, and/or sequencing of individual clones, followed by low-throughput western-blotting to identify the expressed protein (Neupert et al. 2009; Georgianna et al. 2013; Soria-Guerra et al. 2014). Additionally, exonuclease activity can degrade transformed DNA, leading to truncated or damage transgenes before the exogenous DNA integrates into the genome (Jinkerson and Jonikas 2015). This can result in drug-resistant transformants that do not even contain a full recombinant protein gene linked to the drug resistance gene. Depending on vector design, these truncation events represent a substantial percentage of transformants (Gonzalez-Ballester et al. 2011; Meslet-Cladière and Vallon 2011; Zhang et al. 2014), if not a majority of the recovered antibiotic resistant clones (Li et al. 2016). Since at best, < 1% of algae cells result in successful transformants (Life Technologies Corporation 2013), this means thousands or millions of algae cells need to be screened per transformation to obtain clones with the highest protein expression. Therefore, improving high-throughput screening (HTS) methods for the detection of transgenic cells expressing desired proteins is necessary to advance algae as an efficient system for recombinant protein production.

Previously, most HTS methods for microalgae were developed for the identification of high-lipid producing strains for purposes of producing algae-based biofuels (Xie et al. 2014; Zhang et al. 2014). Nile Red and BODIPY stains are commonly used to fluorescently label algal lipids, allowing clones to then be analyzed using fluorescence measurements through flow cytometry, fluorescence-activated cell sorting (FACS), or fluorescent microplate reader assays (Mendoza et al. 2008; Chen et al. 2009; Montero et al. 2011; Pereira et al. 2011; Cagnon et al. 2013; Velmurugan et al. 2013; Xie et al. 2014; Terashima et al. 2015; Yamada et al. 2016; Smalley et al. 2020). More recently, these methods have been employed to examine *Chlamydomonas* populations for expression of fluorescent reporter proteins as proxy for recombinant protein expression (Rasala et al. 2013; Scranton et al. 2016; Fields et al. 2019). FACS is one of the methods capable of isolating individual cells of interest for further study; however, some reports show that cultures screened through FACS can experience poor survival due to shear stress on the cells, and those surviving may not always be high-expressing clones due to sorting errors from the instrument (Velmurugan et al. 2013; Fields et al. 2019). Therefore, HTS processes for identification of recombinant proteins in microalgae need to be optimized by re-considering vector design, improved antibiotic screening, and better FACS instrument settings, adapted specifically to algal cells.

Here, we describe the design of a double-antibiotic selection vector for the expression of recombinant proteins in *Chlamydomonas*, that when coupled with antibiotics and FACS screening is able to quickly identify and isolate large populations of *Chlamydomonas* transformants expressing a fluorescent reporter protein. The use of this new vector and HTS process is highly improved over older methods and can be utilized by the broader research community to enhance screening efficiency, thereby speeding up the discovery and development of algal-derived products.

## Materials and methods

### Vector design and cloning

**Fig. 1:**
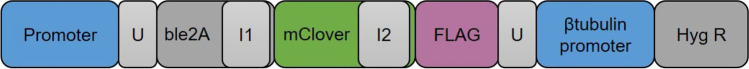
Design of double-antibiotic resistance expression vector. The variable “Promoter” region is upstream of the *rbcs2* 5′ and 3′ UTRs (U). The *ble2A* gene for zeocin resistance is fused to an mClover reporter with the first (I1) and second (I2) *rbcs2* introns. A FLAG tag is included upstream of a beta-tubulin promoter driving the hygromycin resistance cassette. Figure created with Microsoft Office

To address the problem of truncated transgenes and low efficiency of recovered gene-of-interest–expressing clones, an expression vector was developed with the gene of interest flanked by two different drug resistance genes: zeocin resistance (*ble2A*) and hygromycin resistance (Fig. 1). The upstream promoter is allowed variability; here we constructed the vector using three different promoters: AR1, a basal *rbcs2* promoter, and SAP11, a synthetic promoter previously found to drive higher expression than AR1 (Scranton et al. 2016). We also constructed a vector without the promoter region to act as a negative control (NC). A FLAG-tagged, modified green fluorescent protein (GFP), mClover, derived from the pOpt series of vectors, is used as the gene-of-interest to act as a reporter since it is one of the brightest and most photostable GFP variants codon optimized for use in *C. reinhardtii* (Lam et al. 2012; Lauersen et al. 2015). Meanwhile, the downstream beta-tubulin promoter drives the hygromycin resistance cassette, a commonly used tool in *Chlamydomonas* expression vectors (Berthold et al. 2002; Lauersen et al. 2015). This design supports a high-throughput process by allowing determination of full-length construct integration by observing growth on antibiotic plates, rather than having to perform lengthy primer walk sequencing of each clone, since clones with truncated transgenes would not be able to grow in the presence of both antibiotics.

Components of the vector were derived from the pOpt vector system (Lauersen et al. 2015) and assembled into the pUC19 backbone (Norrander et al. 1983) through PCR with Q5 DNA polymerase and NEBuilder HiFi DNA Assembly Cloning Kit (New England Biolabs; catalog E5520S). Variations in the promoter region were additionally constructed using the same assembly method. All constructs were confirmed by primer walk sequencing of the full constructs; the full list of primers is included with Online Resource 2. Once the sequences were confirmed, plasmid DNA was linearized by restriction digest with *Kpn*I and *Xba*I (New England Biolabs). The digest was then purified using the Wizard® SV Gel and PCR Clean-Up System (Promega), and DNA was quantified using Qubit dsDNA HS Assay Kit (Thermo Fisher Scientific). The vectors were then transformed into chemically competent *E. coli* cells (5-alpha High Efficiency, New England Biolabs) and verified by Sanger sequencing through the entirety of the *Chlamydomonas*-specific sequences between the M13 Forward and M13 Reverse universal primer sites of the pUC19 vector backbone. Complete vector sequences have been submitted to NCBI (GenBank Accession #: OK247606 (pAES8; SAP11), OK247607 (pAES7; no promotor), OK247608 (pAES6; *rbcs2*), OK247609 (pAES5; AR1).

### Transformation into Chlamydomonas reinhardtii

Cultures of wild-type (WT) *C. reinhardtii* strain CC125 (obtained from chlamycollection.org) were grown and transformed via electroporation using modified methods of Rasala et al. (2012). Cells were grown under 100 μmol m^−2^ s^−1^ light and maintained at 25 °C on a shaker table at 125 RPM. Once cells reached 0.5–1 × 10^6^ cells/mL, they were harvested by resuspending to 1–2 × 10^8^ cells/mL in GeneArt® MAX Efficiency® Transformation Reagent (Invitrogen). For each vector, 2 μg linearized DNA was added to 250-μL algae cells and transferred to a 4-mm cuvette, then incubated on ice for 5 min. An electric pulse of 500 V/cm was applied to each sample using a Gene Pulser Xcell™ Electroporation System (Bio-Rad). The capacitance was set at 50 μF and resistance at 800 Ohm. Cuvettes were immediately transferred to a 25 °C-water bath for 15 min, and then recovered for 18 h in 10 mL of TAP (Tris–acetate-phosphate) + 40 mM sucrose under lowered light conditions (~ 85 μmol m^−2^ s^−1^).

### Screening with antibiotic selection

The entire population of algae transformants were initially screened by plating a 1:20 diluted culture on TAP/agar plates containing a single antibiotic (30 μg/mL hygromycin) and plating undiluted culture on TAP/Agar plates containing two antibiotics (30 μg/mL hygromycin + 15 μg/mL zeocin), to compare differences between single (Hyg) and double (Hyg + Zeo) antibiotic selection. Plating was designed this way to plate entire transformation between both treatments while maintaining well-separated colony growth, based on previous unpublished observations of colony frequencies on each plate type.

### Screening with fluorescence-activated cell sorting (FACS)

After 9 days of growth under 100 μmol m^−2^ s^−1^ light, antibiotic plates had formed algal colonies that were scraped into 50-mL sterile tubes containing 20 mL TAP using a rubber spatula. The liquid cultures were incubated under the same light conditions on shaker table for 4 days to allow cell proliferation for screening with FACS.

One day prior to analysis with FACS, algae cultures were passed through 40-μm cell strainers into new sterile 50-mL tubes to remove any agar debris and break-up cell clumps. One hour prior to analysis with FACS, 3 mL of each culture was added to a 5-mL round bottom polystyrene test tube with 35-μm cell strainer cap. A Beckman Coulter MoFlo Astrios with a 100-um nozzle and BioSure® Preservative-Free Sheath Solution was used to collect flow cytometry (FC) data and sort algae cells. This analysis utilized a 488-nm laser with SSC and FSC detection, as well as a 513/26 (FL10) filter to detect mClover fluorescence and a 710/45 (FL9) to detect chlorophyll autofluorescence. To identify transgenic cells positive for the mClover reporter protein, a sample of *C. reinhardtii* CC125 was analyzed and used to create a gate for the mClover negative ( −) population. A gate was then placed further along the mClover axis to identify cells with fluorescence higher than WT, which indicate they are positive ( +) for the reporter protein.

The top 10% of the mClover + cell populations were sorted into six-well plates filled with 3 mL TAP per well. Number of cells sorted ranged from 1.9 to 10 K and was dependent on population density (Table 1). Cells were grown in the plates under the same light conditions for 4 days, and then FC data was obtained for each enriched population and analyzed using FlowJo X. Cells within the mClover + gate were exported as individual events, and the mClover fluorescence was normalized to chlorophyll to give relative fluorescence units (RFU). An unpaired two-tailed *t* test was performed using Microsoft Excel version 2019, to compare RFU between Hyg and Hyg + Zeo populations for each vector, both before and after FACS enrichment. Ten thousand cells from the mClover + populations of the enriched samples were sorted into new 50-mL culture tubes containing fresh TAP, allowed to grow for 1 week, then divided into aliquots that were either re-plated on hygromycin for colony picking, or pelleted and stored in 1-mL fractions at − 80 °C to use for DNA extraction and Western blots.

**Table 1:** Resulting colonies of algal transformants per construct, after 9 days of growth on agar plates containing a single antibiotic (hygromycin only; Hyg) and two antibiotics (hygromycin + zeocin; Hyg + Zeo)

Vector	Total colonies on Hyg (% mClover +)	Total colonies on Hyg + Zeo (% mClover +)	Hyg:Hyg + Zeo colony ratio
NC	1672 (0.3%)	18 (85.2%)	4180:1
rbcs2	840 (2.7%)	268 (62.9%)	160:1
AR1	2116 (3.1%)	310 (62.7%)	272:1
SAP11	1776 (3.4%)	193 (61.0%)	470:1

### Verification of recombinant mClover

#### Fluorescence measurements

Transgenic algae containing the AR1 construct was selected for visualization of recombinant GFP, since it is the most commonly used promoter in *Chlamydomonas* research and can be used as a control in further studies. After re-plating the enriched cultures, 24 of the resulting single colonies were randomly picked into 96-well plates filled with 150 μL TAP media along with 24 colonies of the CC125 strain for reference. Cultures were incubated in the light for 3 days, then sub-cultured into new plates to remove cell clumps, and analyzed with a TECAN plate reader. Fluorescence of mClover was measured at an excitation/emission wavelength of 505/540 nm and was normalized by chlorophyll measurements at 440/680 nm as shown in Online Resource 1. Fold change of mClover over WT algae was calculated, and one high-expressing clone (~ 8X higher mClover than WT, called AR1_D12) capable of long-term survival on archive plates was used for imaging. Differential interference contrast (DIC) and fluorescence microscopy were performed using a DeltaVision Core system (Applied Precision) composed of an Olympus IX71 inverted microscope equipped with Olympus UPlanSApo 100 × /1.40 objective and 1.6 × auxiliary magnification enabled, with a CoolSNAP HQ2/ICX285 camera. Tetramethylrhodamine isothiocyanate (TRITC) filters (EX555/28 and EM617/73) were used to image autofluorescence of photosynthetic pigments, while the GFP (EX470/40 and EM525/36) filters were used to image heterologous protein fluorescence. Image acquisition, deconvolution, and analysis were performed using Resolve3D softWoRx-Acquire (Version 5.5), and ImageJ was used for brightness/contrast adjustments and figure panel construction. Exposure times were consistent for all samples as follows: DIC = 50% transmission, 0.4-s exposure, GFP = 100% transmission, 0.3-s exposure, TRITC = 100% transmission, 2-s exposure.

#### DNA extraction and sequencing

Cells sorted from the enriched culture were grown in the light for 4 days, then 1 mL of algae from each culture was centrifuged for 5 min at 5000 × *g* at room temperature (RT). The supernatant was removed, and the algal pellet was used for genomic DNA extraction by adding 650 μL of warm (65 °C) LDS lysis buffer (1% lithium dodecyl sulfate; 20 mM Tris–HCl, pH 8.0; 2 mM sodium EDTA, pH 8; 500 nM NaCl) and incubating in a 6 °C-water bath for 20 min. 650 µL of phenol:chloroform:isoamyl alcohol pH 8 was added, and lysate was shaken to emulsion for ~ 10 s. Samples were spun at 5000 × *g* for 4 min at room temperature (RT), then 500 μL of the upper aqueous phase was recovered and mixed with 750 μL of ethanol-stabilized chloroform. After centrifugation at 16,000 × *g* for 1 min at RT, 400 μL of the upper aqueous phase was recovered and washed with 750 μL ethanol-stabilized chloroform for a second time. 300 µL of the aqueous phase was then recovered and 1 μL of 5 mg/mL linear polyacrylamide was added and vortexed to mix. Next, 300 μL of isopropanol was added to precipitate DNA, and samples were centrifuged for 30 min at 16,000 × *g* for 30 min. Once the supernatant was removed, the DNA pellet was washed twice with 1 mL of 70% ethanol. DNA was then dissolved in 150 μL of 1X TE pH 8. DNA concentration was quantified with Qubit dsDNA HS Assay Kit (Thermo Fisher Scientific) then amplified with PCR.

Q5 Hot-Start Polymerase (New England Biolabs) was used to amplify vectors from algal genomic DNA in 50-μL reactions. Thermocycling was conducted with initial denaturation at 98 °C for 30 s, followed by 35 cycles of 98 °C for 10 s, 62 °C for 20 s, 72 °C for 20 s, with final extension at 72 °C for 2 min, and an indefinite hold at 4 °C. Primers were designed to amplify only the variable promoter region of each vector. The forward primers AES51_F1 (AATTCGCGATTATAACGGC), AES61_F1 (AATTCGCGATTATAACCGG), AES71_F1 (AATTCGCGATTATAAGAGAAGTC), and AES81_F1 (AATTCGCGATTATAACACATGC) were used to amplify AR1, rbcs2, NC, and SAP11 vectors, respectively. The reverse primer AES51_R1 (GGTCAGCTTGGCCAT) was used to amplify all promoters. Purified PCR products were sequenced using AES910_F1 (ATTTAAATTCGCGATTATAA) and AES51_R1 10 mM primers, and resulting sequences were assembled to the reference vectors using DNAbaser software.

#### Western blotting

Frozen pellets of sorted transgenic algae populations containing each construct were thawed, then resuspended and lysed in 1X BugBuster Protein Extraction Reagent with 0.06% Benzonase® endonuclease for 10 min on ice. Lysate was centrifuged at 5000 × *g* for 5 min at 4 °C to pellet cell debris, then supernatant was recovered as clarified lysate. Total soluble protein (TSP) concentration was measured using the Thermo Scientific™ Coomassie (Bradford) Protein Assay Kit as per the manufacturer’s instructions. Lysates were then normalized to 3 μg TSP and mixed with 4 × Laemmli buffer with 10% vol/vol β-mercaptoethanol. Samples were heated in an 80 °C-water bath for 10 min then allowed to cool. Proteins were separated by SDS-PAGE on 12% Mini-PROTEAN® TGX™ Precast Protein Gels at 200 V, then transferred onto nitrocellulose membrane at 15 V for 1 h. After blocking with TBSMT + 1% PVP-40 (Haycock 1993), membranes were probed with an anti-GFP monoclonal antibody conjugated to alkaline phosphatase (abcam, ab6661). The AccuRuler RGB Plus protein marker was used for reference band sizes, while wild-type *C. reinhardtii* strain CC125 was used as a negative control, and the high GFP-expressing single clone from the AR1 construct was used as a positive control.

## Results

**Fig. 2:**
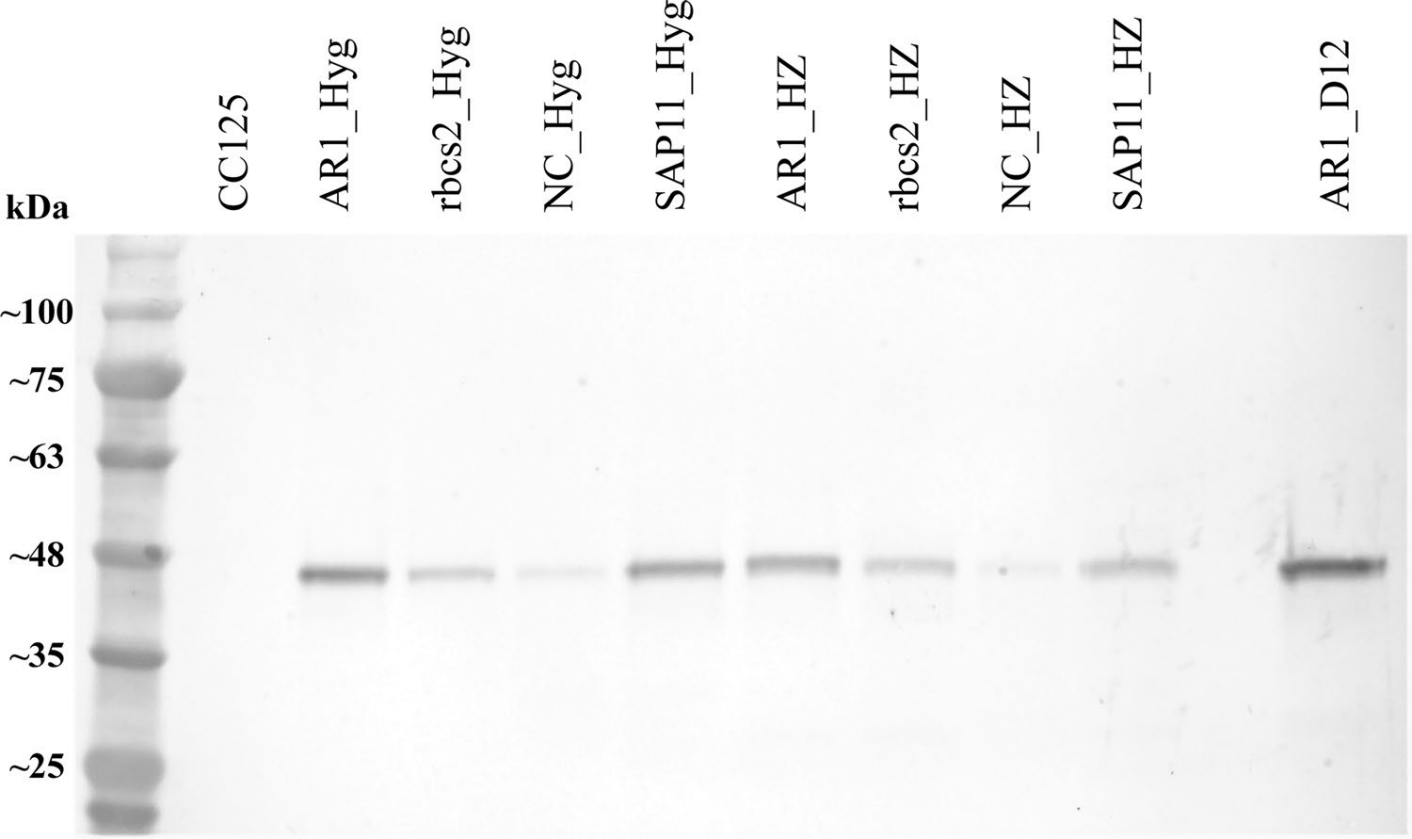
Western blot of transgenic algae cultures after FACS enrichment, compared to WT CC125 strain and high GFP-expressing clone AR1_D12 confirmed via microscopy. Hyg, hygromycin screened only, HZ hygromycin + zeocin screened. The mClover-Ble protein is ~ 41 kDa

**Fig. 3:**
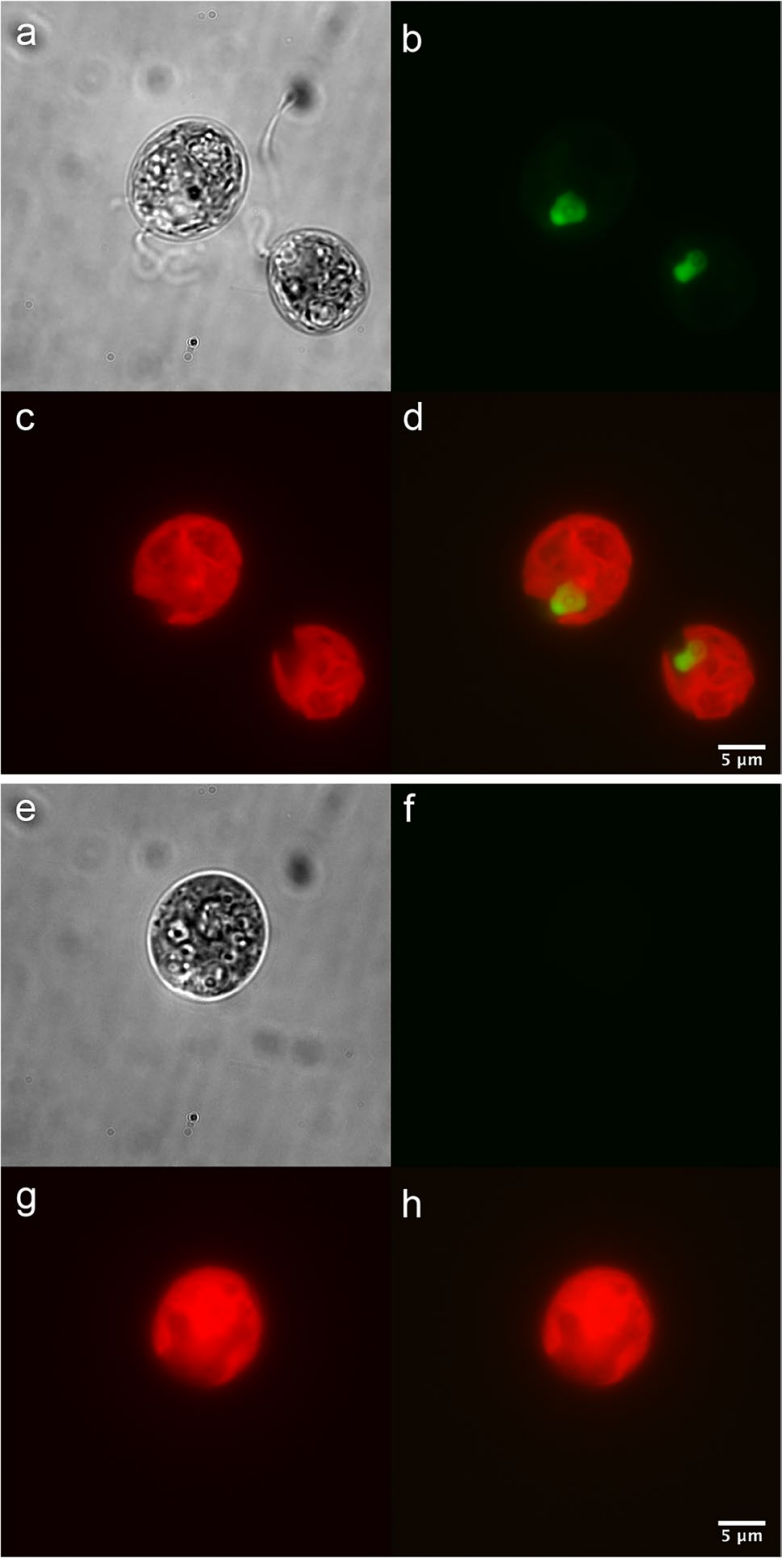
DIC and fluorescence microscopy images of transgenic *C. reinhardtii*, clone AR1_D12, expressing mClover in the nucleus (**a**–**d**) compared to wild-type *C. reinhardtii* strain CC125 (**e**–**h**). **a**, **e** Cells observed under DIC. **b**, **f** Cells observed under GFP filters. **c**, **g** Chlorophyll autofluorescence of cells, observed under TRITC filters. **d**, **h** Merging of GFP and chlorophyll channels. GFP channels were all set to the same contrast (bracketed on raw pixel values of 50–2000) for both transgenic and WT strains, while the chlorophyll channel was similarly bracketed for all images (50–250 raw pixel values). Imaging performed by Ryan Simkovsky (UCSD)

Between 900–2000 transformants from Hyg selection and 10–100 transformants from Hyg + Zeo selection were harvested from each of the four different constructs for initial flow cytometry analysis (Table 1). Higher colony frequencies were observed on Hyg plates compared to Hyg + Zeo plates for each construct, as expected. DNA sequencing of the sorted cell populations confirmed that the transformants had the intended sequences after FACS enrichment, with the NC vector containing no PCR product. Western blot analysis showed that all enriched transgenic algae cultures were producing recombinant mClover, which has a size of ~ 41 kDa when fused with Ble, with the NC having only a faint signal under both antibiotic treatments (Fig. 2). Expression of the fluorescent reporter protein can also be confirmed visually in the nucleus when observed by fluorescence microscopy (Fig. 3).

**Fig. 4:**
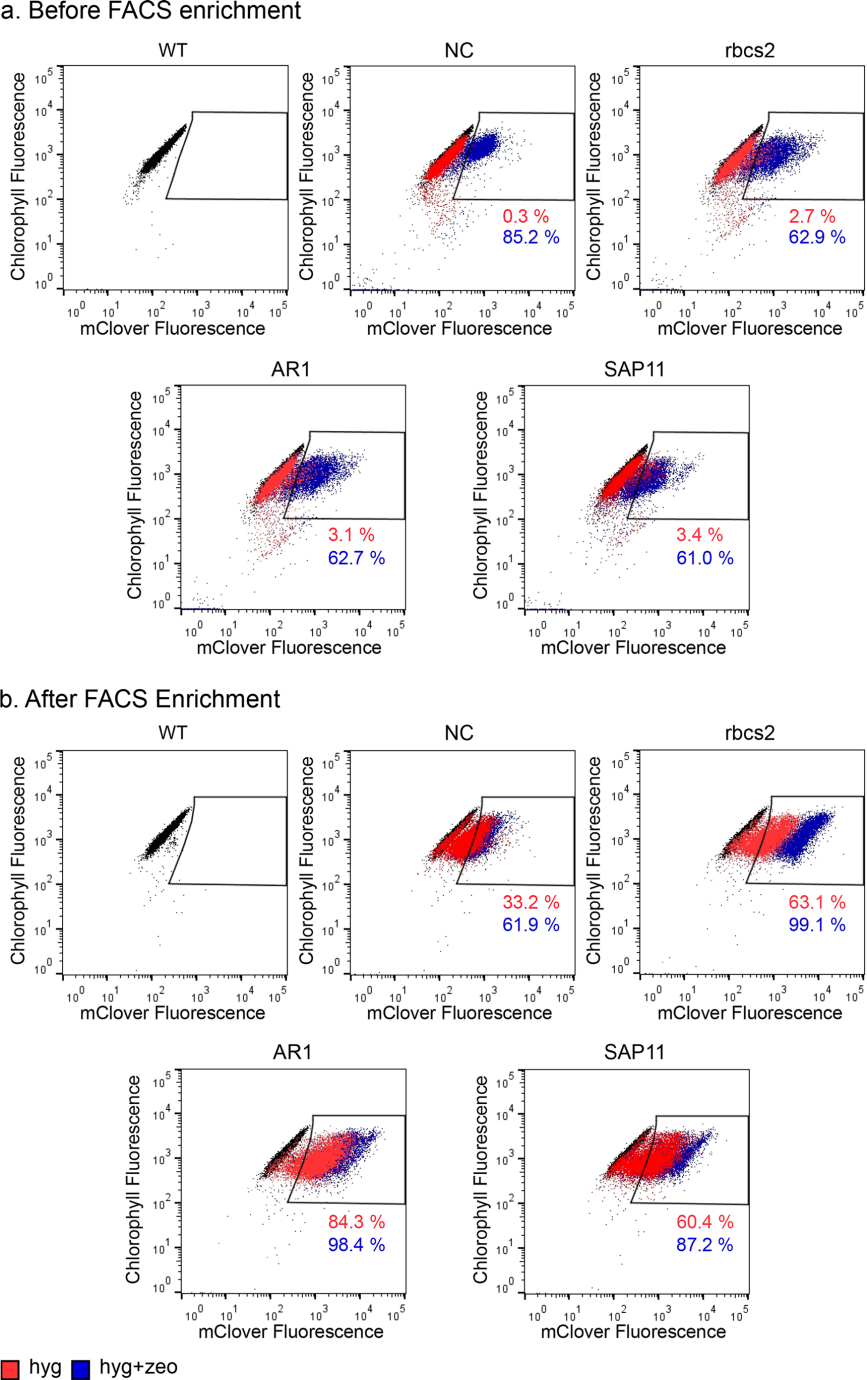
Effect of FACS enrichment on mClover + cell populations resulting from expression vectors with variable promoter regions. Algae transformants were screened on agar plates containing either hygromycin (red) or hygromycin + zeocin (purple), then surviving colonies were inoculated into liquid culture and analyzed by flow cytometry **a** 4 days after initial inoculation and **b** 4 days after sorting the top 10% of the mClover + population into fresh TAP media. Black populations in each plot show the baseline measurements for wild type (WT) *C. reinhardtii* CC-125. Gates higher on the mClover axis designate populations with mClover + expression. Percentages under gates correspond with the amount of each population that is contained within the mClover + gate. Figure created with FlowJo X and Inkscape

The first round of FACS revealed that hyg transformants had a < 4% mClover-positive (mClover +) population, while hyg + zeo transformants had between 16 and 29% mClover + populations (Table 1, Fig. 4). These values increased after FACS enrichment up to 63% in Hyg transformants and 99% in Hyg + Zeo transformants (Fig. 4).

**Fig. 5:**
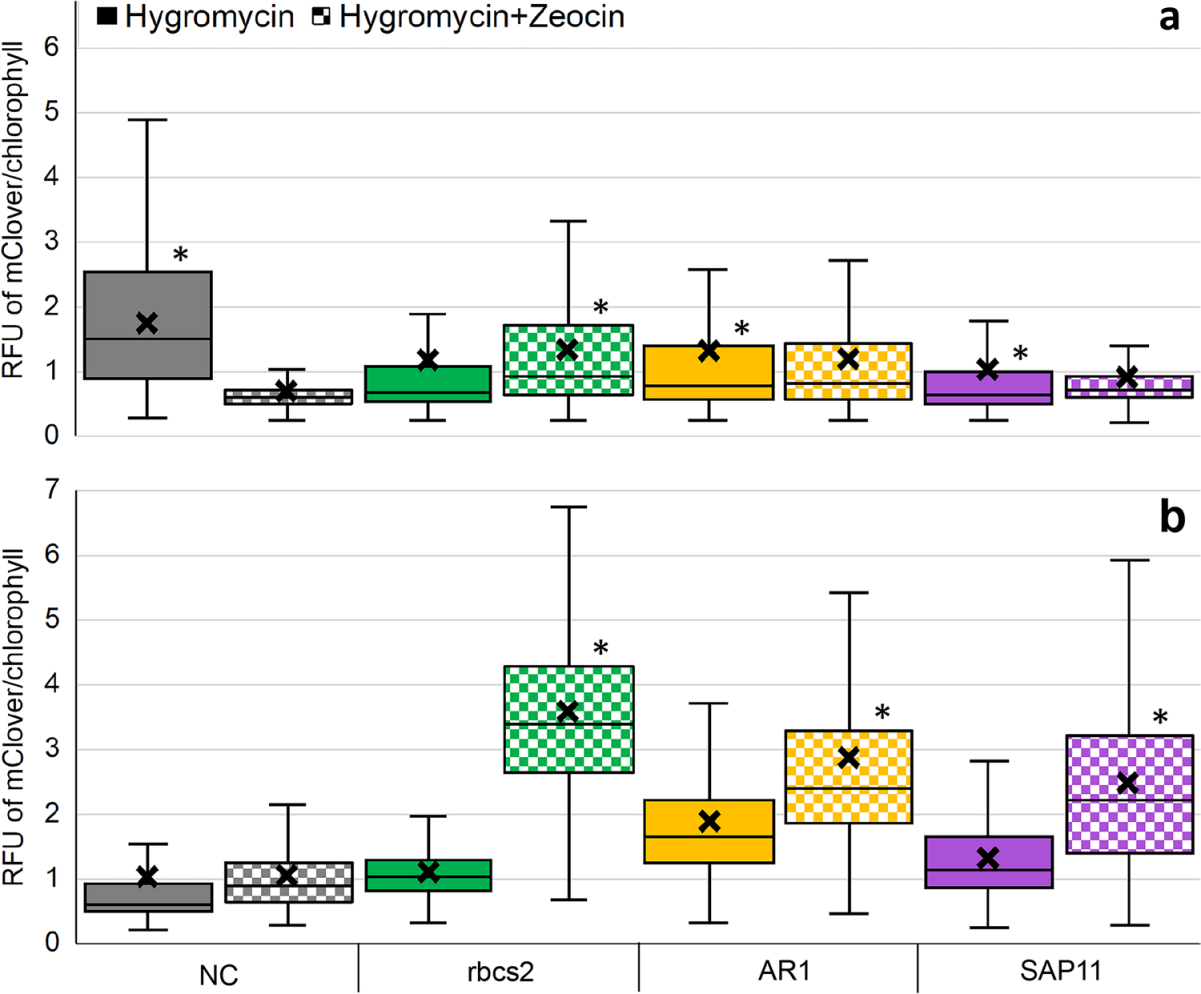
Flow cytometry data examining the relative fluorescence units (RFU) of mClover normalized to chlorophyll fluorescence in *C. reinhardtii*. **a** After transformation with either the NC, rbcs2, AR1, or SAP11 vector, cells were recovered on agar plates with either hygromycin (solid boxes) or hygromycin + zeocin (checkered boxes) and then examination via flow cytometry. The distribution of the RFU from these initial transformants includes those whose mClover fluorescence was in the region above wild-type autofluorescence. Cells fluorescing in the top 10% of this region were isolated via fluorescence-activated cell sorting (FACS). **b** After FACS, cells were again recovered on agar plates with either hygromycin (solid boxes) or hygromycin + zeocin (checkered boxes) and then examination via flow cytometry for a second time. *N* = 2000 per box plot, *x* indicates the mean, and asterisks indicate any significant difference (*p* < 0.01) between mean RFU when comparing antibiotic treatment within respective colored pairs. Figure created with Microsoft Office

The relative fluorescence of mClover + cells was significantly different between hyg and hyg + zeo-screened transformants prior to FACS enrichment for all vectors (*p* < 0.01), with only the rbcs2 vector having higher expression in the hyg + zeo vs the hyg population (Fig. 5). However, after FACS enrichment, all of the vectors other than the NC had significantly higher mClover fluorescence (*p* < 0.01) in the hyg + zeo populations (Fig. 5). With the NC vector being a negative control lacking a promoter to drive the *ble2a* gene, its inability to be enriched is not surprising. The promoter-less vector was used here as a control, where expression of the gene would result only if the cassette landed in a region of the genome that could support transcription of the recombinant gene. This is not expected to be a common event, and hence the resulting low number of colonies on hyg + zeo plates was expected.

## Discussion

The higher RFU of the hyg + zeo-screened transformants only after FACS enrichment shows that the double antibiotic screen more efficiently isolates clones with higher expression of mClover. Three of the vectors resulted in hyg-screened transformants with higher mClover RFU than hyg + zeo transformants before FACS enrichment; therefore, sorting for the top 10% of these populations should have resulted in higher RFU in the enriched culture as well. However, after FACS enrichment, all the promoter-containing vectors experienced higher mClover RFU in the hyg + zeo-screened populations. This indicates that the transformants sorted from the hyg mClover-positive population may have contained truncated transgenes, or perhaps these clones lost mClover expression over time due to rapid gene silencing. Previous studies have found that *C. reinhardtii* transgenes commonly become silenced from 20 to 200 days after transformation (Koblenz et al. 2003; Iomini et al. 2006). Yamasaki et al. (2008) found that this silencing is due to epigenetic effects that evolved in the algae to protect against viruses and transposable elements, and that subclones can fluctuate in their degree of transgene silencing throughout mitotic cell division. Therefore, our selection method is potentially screening out the transformants with less epigenetic silencing. Additionally, the expression of the hygromycin resistance gene has been observed as extremely unstable even when the associated exogenous gene expression is not (Ladygin and Boutanaev 2002), and could explain why the addition of a second antibiotic resistance gene provides better results.

The synthetic algae promoter, SAP11, was previously shown to drive expression of a fluorescent reporter protein up to 2 × higher than that of AR1 (Scranton et al. 2016). However, when used in our HTS process, it did not result in as many mClover + transformants as AR1 after FACS enrichment, nor did it drive mClover fluorescence to a degree visibly higher than AR1 (Figs. 4 and 5). In fact, we found that even the *rbcs2* core promoter alone resulted in more mClover + transformants with higher mClover RFU than SAP11 after FACS enrichment of the hyg + zeo-screened algae population. Differences may be due to the initial study using a different vector design and *C. reinhardtii* background strain, as well as not conducting FACS enrichment. This synthetic promoter may be less effective at driving recombinant protein expression under these experimental conditions, and follow-up experiments with SAP11 should be conducted to determine its functionality in different strains and vector designs. Interestingly, the differences are not as apparent in the hyg-screened transformants, further demonstrating the utility of the double selection vector in providing more cells for fluorescence assessments, leading to more robust comparisons of vector strengths.

The design of optimized expression vectors for protein expression is critical for improving the efficiency of downstream screening methods by providing a larger population of positive transformants to select from. Furthermore, HTS can be a critical step in the development of bioproducts from microorganisms as it reduces the time and costs of identifying strains with the desired phenotype. FACS has recently become an important tool in the microalgal biotechnology field, as it can be used in bioprospecting for novel strains, achieving axenic cultures, and in strain improvement by enrichment of cells with specific properties; however, the majority of previous studies have focused on screening for lipid-rich microalgae, rather than other gene products and biomolecules (Pereira et al. 2018). The technique has more recently been demonstrated for wider applications, such as screening of the microalga *T. lutea*, which allowed production of the valuable compounds fucoxanthin (Fx) and docosahexaenoic acid (DHA) to reach industrial levels at outdoor pilot scale after only two rounds of FACS (Gao et al. 2020; Gao et al. 2021). Continued development of FACS methods for screening improved algal strains in this manner will more rapidly advance the field of microalgal biotechnology for a larger diversity of commercial bioproducts.

In more developed recombinant protein production platforms, such as bacteria, screening of clones using antibiotic resistance genes, as well as HTS using fluorescent reporter fusion proteins or fluorescent probes with FACS, has long been utilized to monitor and isolate desired overexpression phenotypes (Makino et al. 2011). In fact, when using GFP as a reporter protein in *E. coli*, it was found that fluorescence correlated well with robust protein folding and expression levels of fused recombinant proteins (Waldo et al. 1999). In these systems, FACS has been used to successfully improve transformant screening for desired targets in multiple different ways. One interesting method for screening enzyme gene libraries is through compartmentalizing either recombinant single cells, or cell-free single gene copies, into aqueous water droplets that are then emulsified within oil droplets with a fluorogenic substrate; the enzyme reacts with the substrate and is then double-emulsified into a water-in-oil-in-water microdroplet that is then sorted via FACS to isolate and enrich for high enzyme activity (Aharoni et al. 2005; Mastrobattista et al. 2005). Additionally, protein libraries generated using display technologies can also be screened using FACS to link the protein function to the gene encoding that protein. This is achieved through engineering the target protein as fused to a carrier protein that will display it on the cell’s outer surface, where it can be labelled with a fluorescent-conjugated ligand and sorted for binding affinity (Daugherty 2007). These methods could be considered as potential avenues to explore enzyme engineering in microalgae as well.

**Fig. 6:**
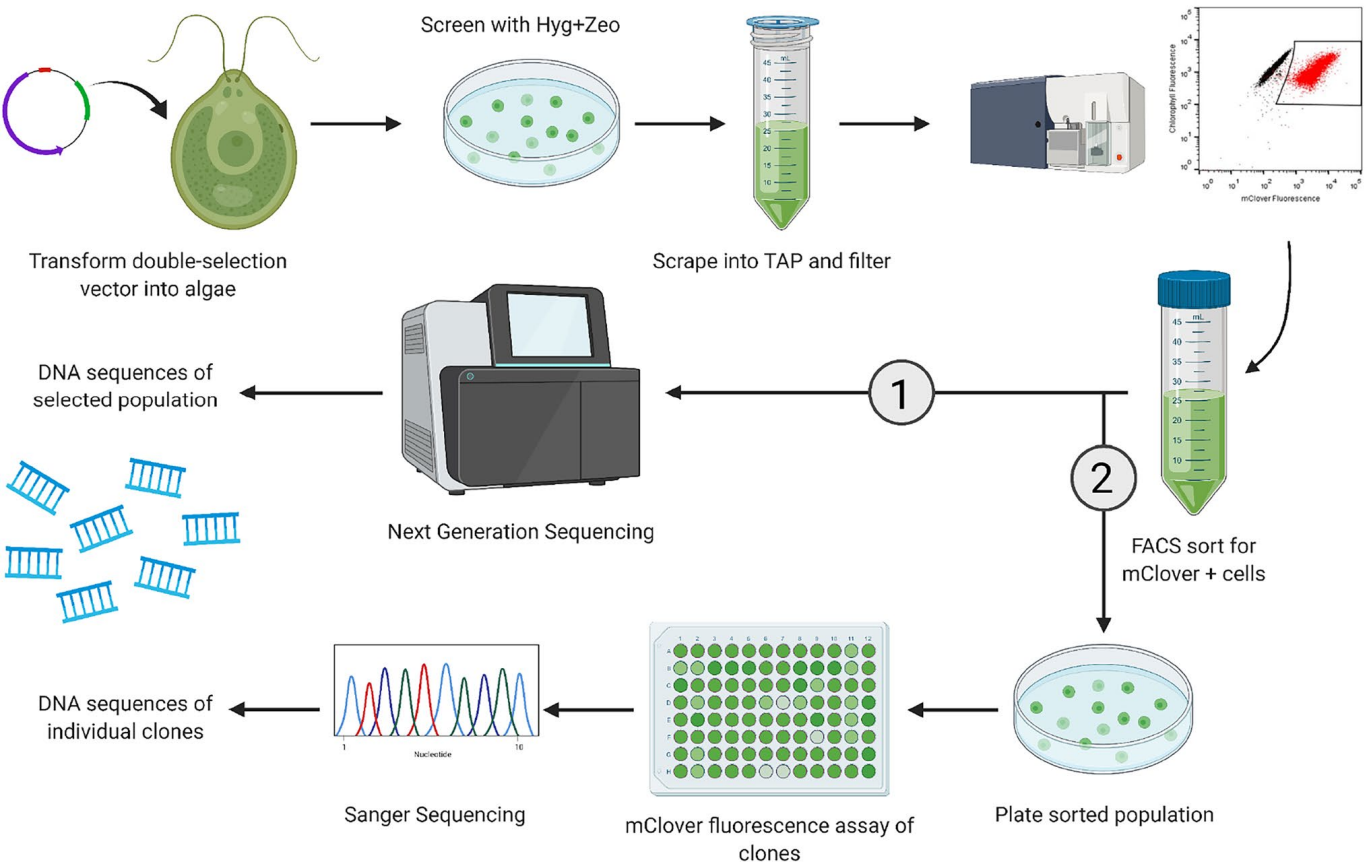
Overview of new high-throughput screening process using double antibiotic selection and FACS. An expression vector containing an mClover fluorescent reporter protein flanked by zeocin and hygromycin resistance genes is transformed into algae, then screened on agar plates containing both antibiotics. Resulting colonies are inoculated into liquid TAP culture and high-expressing cells are sorted by FACS. The enriched culture can be used for (1) whole population sequencing to detect genetic variability or (2) re-plated for picking colonies into microplates for individual fluorescence measurements and sequencing of clones of interest. Figure created with Biorender.com

In conclusion, this study describes both an improved expression cassette, and high-throughput screening process, that allows for the rapid identification of robust recombinant protein expression in the green algae *Chlamydomonas reinhardtii.* Utilizing a double-antibiotic-resistance selection vector, coupled with low pressure FACS, allows for increased post-sort survival of transformants with a high percentage of recombinant protein expression. The process outlined here is able to produce large populations of successful algae transformants with nearly 100% displaying expression of the protein of interest, in as little as three weeks. These cell populations can then be used for applications such as DNA sequencing of the entire enriched population, which would be especially helpful when screening algae populations containing genetic libraries with next-generation sequencing, and they can be re-plated for picking single colonies for individual clone analysis (Fig. 6). Previous studies have used FACS to screen microalgae for desired phenotypes, but generally result in low yields of positive populations (< 1%) that results in a more low-throughput and error-prone output (Xie et al. 2014; Fields et al. 2019). Here, we demonstrated that utilizing double selection with both zeocin and hygromycin, as opposed to hygromycin only, prior to FACS, we were able to significantly increase the percentage of transformants expressing the reporter protein from less than 5% to over 60%. Only one round of FACS enrichment was required to improve the reporter expression to nearly 100% of transformants in the hyg + zeo-screened populations. Standardization of flow cytometric methods and rapid identification of the desired traits will be essential to developing feasible bioprocesses in any algae production platform (Hyka et al. 2013). To further advance the development of high-value recombinant proteins in microalgae, the optimized HTS process we describe here can be utilized and adapted by other research groups studying recombinant proteins in microalgae to identify and isolate large populations of successful transformants, ideally demonstrating the versatility of the method with various background strains and target products.

## Supplementary Information

Below is the link to the electronic supplementary material.

Supplementary file1 (XLSX 18 KB)

## Data Availability

All data generated or analyzed during this study are included in this article and associated supplementary information files.

## References

[CR1] Aharoni A, Amitai G, Bernath K, Magdassi S, Tawfik DS (2005). High-throughput screening of enzyme libraries: thiolactonases evolved by fluorescence-activated sorting of single cells in emulsion compartments. Chem Biol.

[CR2] Berthold P, Schmitt R, Mages W (2002). An engineered *Streptomyces hygroscopicus* aph 7″ gene mediates dominant resistance against hygromycin B in *Chlamydomonas reinhardtii*. Protist.

[CR3] Borowitzka MA (2013). High-value products from microalgae-their development and commercialisation. J Appl Phycol.

[CR4] Cagnon C, Mirabella B, Nguyen HM, Beyly-Adriano A, Bouvet S, Cuiné S, Beisson F, Peltier G, Li-Beisson Y (2013). Development of a forward genetic screen to isolate oil mutants in the green microalga *Chlamydomonas reinhardtii*. Biotechnol Biofuels.

[CR5] Cheah WY, Loke Show P, Chang J-S, Ling C, Juan C (2014) Biosequestration of atmospheric CO2 and flue gas-containing CO2 by microalgae. 10.1016/j.biortech.2014.11.026 10.1016/j.biortech.2014.11.026 25497054

[CR6] Chen W, Zhang C, Song L, Sommerfeld M, Hu Q (2009). A high-throughput Nile red method for quantitative measurement of neutral lipids in microalgae. J Microbiol Methods.

[CR7] Chew KW, Yap JY, Show PL, Suan NH, Juan JC, Ling TC, Lee DJ, Chang JS (2017). Microalgae biorefinery: high value products perspectives. Bioresour Technol.

[CR8] Daugherty PS (2007). Protein engineering with bacterial display. Curr Opin Struct Biol.

[CR9] de Carpentier F, Le Peillet J, Boisset ND, Crozet P, Lemaire SD, Danon A (2020). Blasticidin S Deaminase: A new efficient selectable marker for *Chlamydomonas reinhardtii*. Front Plant Sci.

[CR10] Dismukes GC, Carrieri D, Bennette N, Ananyev GM, Posewitz MC (2008). Aquatic phototrophs: efficient alternatives to land-based crops for biofuels. Curr Opin Biotechnol.

[CR11] Dyo YM, Purton S (2018). The algal chloroplast as a synthetic biology platform for production of therapeutic proteins. Microbiol (united Kingdom).

[CR12] Eichler-Stahlberg A, Weisheit W, Ruecker O, Heitzer M (2009). Strategies to facilitate transgene expression in *Chlamydomonas reinhardtii*. Planta.

[CR13] Fields FJ, Ostrand JT, Tran M, Mayfield SP (2019). Nuclear genome shuffling significantly increases production of chloroplast-based recombinant protein in *Chlamydomonas reinhardtii*. Algal Res.

[CR14] Fields FJ, Lejzerowicz F, Schroeder D, Ngoi SM, Tran M, McDonald D, Jiang L, Chang JT, Knight R, Mayfield S (2020). Effects of the microalgae *Chlamydomonas* on gastrointestinal health. J Funct Foods.

[CR15] Gao F, Teles (Cabanelas, ITD) I, Ferrer-Ledo N, Wijffels RH, Barbosa MJ (2020) Production and high throughput quantification of fucoxanthin and lipids in *Tisochrysis lutea* using single-cell fluorescence. Bioresour Technol 318:4–11. 10.1016/j.biortech.2020.124104 10.1016/j.biortech.2020.124104 32942095

[CR16] Gao F, Sá M, Cabanelas ITD, Wijffels RH, Barbosa MJ (2021). Improved fucoxanthin and docosahexaenoic acid productivities of a sorted self-settling *Tisochrysis lutea* phenotype at pilot scale. Bioresour Technol.

[CR17] Georgianna DR, Hannon MJ, Marcuschi M, Wu S, Botsch K, Lewis AJ, Hyun J, Mendez M, Mayfield SP (2013). Production of recombinant enzymes in the marine alga *Dunaliella tertiolecta*. Algal Res.

[CR18] Gonzalez-Ballester D, Pootakham W, Mus F, Yang W, Catalanotti C, Magneschi L, De Montaigu A, Higuera JJ, Prior M, Galván A, Fernandez E, Grossman AR (2011). Reverse genetics in *Chlamydomonas*: a platform for isolating insertional mutants. Plant Methods.

[CR19] Harris EH (2009). The *Chlamydomonas* sourcebook: introduction to *Chlamydomonas* and its laboratory use.

[CR20] Haycock JW (1993). Polyvinylpyrrolidone as a blocking agent in immunochemical studies. Anal Biochem.

[CR21] Herrero M, Ibáñez E (2015). Green processes and sustainability: an overview on the extraction of high added-value products from seaweeds and microalgae. J Supercrit Fluids.

[CR22] Hyka P, Lickova S, Přibyl P, Melzoch K, Kovar K (2013). Flow cytometry for the development of biotechnological processes with microalgae. Biotechnol Adv.

[CR23] Iomini C, Li L, Mo W, Dutcher SK, Piperno G (2006). Two flagellar genes, AGG2 and AGG3, mediate orientation to light in *Chlamydomonas*. Curr Biol.

[CR24] Jinkerson RE, Jonikas MC (2015). Molecular techniques to interrogate and edit the *Chlamydomonas* nuclear genome. Plant J.

[CR25] Kindle KL, Richards KL, Stern DB (1991). Engineering the chloroplast genome: techniques and capabilities for chloroplast transformation in *Chlamydomonas reinhardtii*. Proc Natl Acad Sci.

[CR26] Khoo HH, Koh CY, Shaik MS, Sharratt PN (2013). Bioenergy co-products derived from microalgae biomass via thermochemical conversion—life cycle energy balances and CO2 emissions. Bioresour Technol.

[CR27] Koblenz B, Schoppmeier J, Grunow A, Lechtreck KF (2003). Centrin deficiency in *Chlamydomonas* causes defects in basal body replication, segregation and maturation. J Cell Sci.

[CR28] Kwon KC, Lamb A, Fox D, Porphy Jegathese SJ (2019). An evaluation of microalgae as a recombinant protein oral delivery platform for fish using green fluorescent protein (GFP). Fish Shellfish Immunol.

[CR29] Ladygin VG, Boutanaev AM (2002). Transformation of *Chlamydomonas reinhardtii* CW-15 with the hygromycin phosphotransferase gene as a selectable marker. Russ J Genet.

[CR30] Lam A, St-Pierre F, Gong Y, Marshall JD, Cranfill PJ, Baird MA, McKeown MR, Wiedenmann J, Davidson MW, Schnitzer MJ, Tsien RY, Lin MZ (2012). Improving FRET dynamic range with bright green and red fluorescent proteins. Nat Methods.

[CR31] Lauersen KJ, Kruse O, Mussgnug JH (2015). Targeted expression of nuclear transgenes in *Chlamydomonas reinhardtii* with a versatile, modular vector toolkit. Appl Microbiol Biotechnol.

[CR32] León-Bañares R, González-Ballester D, Galván A, Fernández E (2004). Transgenic microalgae as green cell-factories. Trends Biotechnol.

[CR33] Li X, Zhang R, Patena W, Gang SS, Blum SR, Ivanova N, Yue R, Robertson JM, Lefebvre PA, Fitz-Gibbon ST, Grossman AR, Jonikas MC (2016). An indexed, mapped mutant library enables reverse genetics studies of biological processes in *Chlamydomonas reinhardtii*. Plant Cell.

[CR34] Life Technologies Corporation (2013) GeneArt ® MAX efficiency ® Transformation reagent for algae: user manual. ThermoFisher Scientific. Publication Number MAN0009795

[CR35] Lingg N, Zhang P, Song Z, Bardor M (2012). The sweet tooth of biopharmaceuticals: importance of recombinant protein glycosylation analysis. Biotechnol J.

[CR36] López Barreiro D, Samorì C, Terranella G, Hornung U, Kruse A, Prins W (2014). Assessing microalgae biorefinery routes for the production of biofuels via hydrothermal liquefaction. Bioresour Technol.

[CR37] Makino T, Skretas G, Georgiou G (2011). Strain engineering for improved expression of recombinant proteins in bacteria. Microb Cell Fact.

[CR38] Manuell AL, Beligni MV, Elder JH, Siefker DT, Tran M, Weber A, McDonald TL, Mayfield SP (2007). Robust expression of a bioactive mammalian protein in *Chlamydomonas* chloroplast. Plant Biotechnol J.

[CR39] Mastrobattista E, Taly V, Chanudet E, Treacy P, Kelly BT, Griffiths AD (2005). High-throughput screening of enzyme libraries: in vitro evolution of a β-galactosidase by fluorescence-activated sorting of double emulsions. Chem Biol.

[CR40] Maul JE, Lilly JW, Cui L, DePamphilis CW, Miller W, Harris EH, Stern DB (2002). The *Chlamydomonas reinhardtii* plastid chromosome: islands of genes in a sea of repeats. Plant Cell.

[CR41] Mayfield SP, Manuell AL, Chen S, Wu J, Tran M, Siefker D, Muto M, Marin-Navarro J (2007). *Chlamydomonas reinhardtii* chloroplasts as protein factories. Curr Opin Biotechnol.

[CR42] Mendoza H, de la Jara A, Freijanes K, Carmona L, Ramos AA, de Sousa DV, Serafim Varela JC (2008). Characterization of Dunaliella salina strains by flow cytometry: a new approach to select carotenoid hyperproducing strains. Electron J Biotechnol.

[CR43] Merchant SS, Prochnik SE, Vallon O, Harris EH, Karpowicz SJ, Witman GB, Terry A, Salamov A, Fritz-Laylin LK, Maréchal-Drouard L, Marshall WF, Qu L-H, Nelson DR, Sanderfoot AA, Spalding MH, Kapitonov VV, Ren Q, Ferris P, Lindquist E, Shapiro H, Lucas SM, Grimwood J, Schmutz J, Grigoriev IV, Rokhsar DS, Grossman AR, Team CA, Team JA (2007). The *Chlamydomonas* genome reveals the evolution of key animal and plant functions. Science.

[CR44] Meslet-Cladière L, Vallon O (2011). Novel shuttle markers for nuclear transformation of the green alga *Chlamydomonas reinhardtii*. Eukaryot Cell.

[CR45] Montero MF, Aristizábal M, García Reina G (2011). Isolation of high-lipid content strains of the marine microalga *Tetraselmis suecica* for biodiesel production by flow cytometry and single-cell sorting. J Appl Phycol.

[CR46] Moreno-Garcia L, Adjallé K, Barnabé S, Raghavan GSV (2017). Microalgae biomass production for a biorefinery system: recent advances and the way towards sustainability. Renew Sustain Energy Rev.

[CR47] Murbach TS, Glávits R, Endres JR, Hirka G, Vértesi A, Béres E, Szakonyiné IP (2018). A toxicological evaluation of *Chlamydomonas reinhardtii*, a Green Algae. Int J Toxicol.

[CR48] Neupert J, Karcher D, Bock R (2009). Generation of *Chlamydomonas* strains that efficiently express nuclear transgenes. Plant J.

[CR49] Neupert J, Shao N, Lu Y, Bock R, Dunwell J, Wetten A (2012). Genetic transformation of the model green alga *Chlamydomonas reinhardtii*. Transgenic plants. Methods in molecular biology (Methods and Protocols).

[CR50] Norrander J, Kempe T, Messing J (1983). Construction of improved M13 vectors using oligonucleotide-directed mutagenesis. Gene.

[CR51] Pereira H, Barreira L, Mozes A, Florindo C, Polo C, Duarte CV, Custádio L, Varela J (2011). Microplate-based high throughput screening procedure for the isolation of lipid-rich marine microalgae. Biotechnol Biofuels.

[CR52] Pereira H, Schulze PSC, Schüler LM, Santos T, Barreira L, Varela J (2018). Fluorescence activated cell-sorting principles and applications in microalgal biotechnology. Algal Res.

[CR53] Potvin G, Zhang Z (2010). Strategies for high-level recombinant protein expression in transgenic microalgae: a review. Biotechnol Adv.

[CR54] Rasala BA, Mayfield SP (2015). Photosynthetic biomanufacturing in green algae; production of recombinant proteins for industrial, nutritional, and medical uses. Photosynth Res.

[CR55] Rasala BA, Lee PA, Shen Z, Briggs SP, Mendez M, Mayfield SP (2012) Robust expression and secretion of Xylanase1 in *Chlamydomonas reinhardtii* by fusion to a selection gene and processing with the FMDV 2A peptide. PLoS One 7(8):e43349 10.1371/journal.pone.0043349 PMC3427385 22937037

[CR56] Rasala BA, Barrera DJ, Ng J, Plucinak TM, Rosenberg JN, Weeks DP, Oyler GA, Peterson TC, Haerizadeh F, Mayfield SP (2013). Expanding the spectral palette of fluorescent proteins for the green microalga *Chlamydomonas reinhardtii*. Plant J.

[CR57] Remacle C, Cardol P, Coosemans N, Gaisne M, Bonnefoy N (2006). High-efficiency biolistic transformation of *Chlamydomonas mitochondria* can be used to insert mutations in complex I genes. Proc Natl Acad Sci.

[CR58] Scranton MA, Ostrand JT, Fields FJ, Mayfield SP (2015). Chlamydomonas as a model for biofuels and bio-products production. Plant J.

[CR59] Scranton MA, Ostrand JT, Georgianna DR, Lofgren SM, Li D, Ellis RC, Carruthers DN, Dräger A, Masica DL, Mayfield SP (2016). Synthetic promoters capable of driving robust nuclear gene expression in the green alga *Chlamydomonas reinhardtii*. Algal Res.

[CR60] Sheehan J, Dunahay T, Benemann J, Roessler P (1998). A look back at the US Department of Energy’s Aquatic Species Program: biodiesel from algae. Natl Renew Energy Lab.

[CR61] Smalley T, Fields FJ, Berndt AJE, Ostrand JT, Heredia V, Mayfield SP (2020). Improving biomass and lipid yields of *Desmodesmus armatus* and *Chlorella vulgaris* through mutagenesis and high-throughput screening. Biomass Bioenerg.

[CR62] Soria-Guerra RE, Ramírez-Alonso JI, Ibáñez-Salazar A, Govea-Alonso DO, Paz-Maldonado LMT, Bañuelos-Hernández B, Korban SS, Rosales-Mendoza S (2014). Expression of an HBcAg-based antigen carrying angiotensin II in *Chlamydomonas reinhardtii* as a candidate hypertension vaccine. Plant Cell Tissue Organ Cult.

[CR63] Specht EA, Mayfield SP (2014). Algae-based oral recombinant vaccines. Front Microbiol.

[CR64] Sproles AE, Fields FJ, Smalley TN, Le CH, Badary A, Mayfield SP (2021). Recent advancements in the genetic engineering of microalgae. Algal Res.

[CR65] Stephens E, Ross IL, King Z, Mussgnug JH, Kruse O, Posten C, Borowitzka MA, Hankamer B (2010). An economic and technical evaluation of microalgal biofuels. Nat Biotechnol.

[CR66] Terashima M, Freeman ES, Jinkerson RE, Jonikas MC (2015). A fluorescence-activated cell sorting-based strategy for rapid isolation of high-lipid *Chlamydomonas* mutants. Plant J.

[CR67] Vahrenholz C, Riemen G, Pratje E, Dujon B, Michaelis G (1993). Mitochondrial DNA of *Chlamydomonas reinhardtii*: the structure of the ends of the linear 15.8-kb genome suggests mechanisms for DNA replication. Curr Genet.

[CR68] Velmurugan N, Sung M, Yim SS, Park MS, Yang JW, Jeong KJ (2013). Evaluation of intracellular lipid bodies in *Chlamydomonas reinhardtii* strains by flow cytometry. Bioresour Technol.

[CR69] Waldo GS, Standish BM, Berendzen J, Terwilliger TC (1999). Rapid protein-folding assay using green fluorescent protein. Nat Biotechnol.

[CR70] Wijffels RH, Barbosa MJ (2010). An outlook on microalgal biofuels. Science.

[CR71] Xie B, Stessman D, Hart JH, Dong H, Wang Y, Wright DA, Nikolau BJ, Spalding MH, Halverson LJ (2014). High-throughput fluorescence-activated cell sorting for lipid hyperaccumulating *Chlamydomonas reinhardtii* mutants. Plant Biotechnol J.

[CR72] Yamada K, Suzuki H, Takeuchi T, Kazama Y, Mitra S, Abe T, Goda K, Suzuki K, Iwata O (2016). Efficient selective breeding of live oil-rich *Euglena gracilis* with fluorescence-activated cell sorting. Sci Rep.

[CR73] Yamasaki T, Miyasaka H, Ohama T (2008). Unstable RNAi effects through epigenetic silencing of an inverted repeat transgene in *Chlamydomonas reinhardtii*. Genetics.

[CR74] Zhang R, Patena W, Armbruster U, Gang SS, Blum SR, Jonikas MC (2014). High-throughput genotyping of green algal mutants reveals random distribution of mutagenic insertion sites and endonucleolytic cleavage of transforming DNA. Plant Cell.

